# Triple Co-infection With Salmonella typhi, Leptospira, and Campylobacter coli in a Patient From Guayaquil, Ecuador: Clinical and Diagnostic Challenges

**DOI:** 10.7759/cureus.73902

**Published:** 2024-11-18

**Authors:** Henry J Parra Vera, Dayci C Buele Chica, Galo G Farfan Cano, Carlos R Cedeño Cevallos

**Affiliations:** 1 Microbiology, Centre for Microbiological Research, Guayaquil, ECU; 2 Biomedicine, Microbiological Research Centre, Guayaquil, ECU; 3 Research, King Juan Carlos University, Móstoles, ESP; 4 Intermediate Care Unit, Hospital General del Norte de Guayaquil Los Ceibos, Guayaquil, ECU; 5 Research, Society of Infectious Diseases of Guayas, Guayaquil, ECU; 6 Medicine, University of Guayaquil, Guayaquil, ECU

**Keywords:** campylobacter coli, campylobacter infections, case reports, enteric fever (typhoid fever), hidden leptospirosis, salmonella typhi

## Abstract

This case report describes the unusual presentation of a 32-year-old male from Guayaquil, Ecuador, who was diagnosed with a rare triple infection caused by *Salmonella typhi*, *Leptospira*, and *Campylobacter coli*. The patient presented with persistent high fever, severe gastrointestinal symptoms, abdominal pain, and jaundice, following the consumption of street food in a resource-limited area. Important clinical findings included hepatosplenomegaly and elevated liver enzymes, which initially complicated the differential diagnosis. Laboratory tests confirmed the presence of all three pathogens, presenting significant diagnostic and therapeutic challenges due to overlapping symptoms and potential antimicrobial resistance (AMR).

The main diagnoses included typhoid fever, leptospirosis, and campylobacteriosis, each requiring distinct yet coordinated treatment approaches. The patient was managed with a combination of antibiotics targeting each pathogen and supportive care to address dehydration and liver dysfunction. After a prolonged hospital stay, the patient recovered with no residual symptoms, underscoring the success of a tailored, multidisciplinary approach in the context of limited healthcare resources.

This case underscores the importance of clinical awareness regarding co-infections, particularly in areas with inadequate sanitation, where infectious diseases are endemic. The successful management of this complex case highlights the necessity of rapid, accurate diagnostics and coordinated therapeutic strategies to improve outcomes in patients with multi-pathogen infections. This report also emphasizes the critical need for surveillance and tailored interventions in regions facing rising AMR, enhancing our understanding of how to approach emerging infectious diseases in underserved populations.

## Introduction

Leptospirosis, a global zoonotic disease caused by *Leptospira* species, affects both animals and humans. The likelihood of host infection depends on *Leptospira*'s ability to survive in the environment, fostering transmission to new hosts [1]. This bacterium often coexists with other pathogens, promoting their growth and contributing to human co-infections. Understanding the dynamics of such transmission is crucial for effective control. In Ecuador, leptospirosis remains an ongoing public health concern. In 2023, the country reported a surge in cases, with 331 confirmed infections by week 20, primarily affecting men aged 20-49. The Guayas province, where Guayaquil is located, consistently exhibits the highest prevalence, highlighting the endemic nature of the disease [1].

*Campylobacter* and *Salmonella* are significant contributors to foodborne illnesses globally [2-7], with antimicrobial resistance (AMR) emerging as a major public health issue. The One Health approach, integrating human, animal, and environmental health, is vital for monitoring zoonotic infections. This strategy enhances our ability to detect and control infections transmitted through food, animals, and the environment.

In Ecuador, *Campylobacter* spp. and *Salmonella typhi* are also endemic, contributing to a significant burden of gastrointestinal diseases [2-9]. *Campylobacter* is a leading cause of bacterial gastroenteritis, particularly in rural areas, with *Campylobacter jejuni* and *Campylobacter coli* being the most common species isolated from both humans and animals, including poultry and dogs. *S. typhi*, the causative agent of typhoid fever, remains a major public health concern, especially in regions with inadequate sanitation [10-13]. Despite their prevalence, diagnostic capabilities in peripheral health units are often limited, resulting in delayed or incomplete identification of these pathogens. Consequently, local health providers must rely on the National Reference Center for public health research to confirm diagnoses. This centralized diagnostic process presents a particular challenge in the management of co-infections, as the complexity of simultaneously diagnosing *Leptospira*, *Campylobacter*, and *Salmonella* can lead to prolonged diagnostic delays, impacting timely treatment decisions. This case from Guayaquil underscores the importance of improving diagnostic infrastructure in regional health facilities to manage such multifactorial infectious diseases more effectively.

In regions with inadequate sanitation, co-infections with enteric pathogens like *Salmonella*, *Campylobacter*, and *Leptospira* are increasingly common, complicating diagnosis and treatment [4,6-8]. A case from Guayaquil, Ecuador, highlights the simultaneous infection of a patient with *S. typhi*, *Leptospira* spp., and *C. coli*, all endemic to the region. These co-infections are concerning due to the prevalence of multidrug-resistant (MDR) *Salmonella* strains and the challenges posed by diagnosing and managing these diseases in an already vulnerable population. Understanding the clinical management of these co-infections is vital for both local populations and international travelers returning from endemic areas.

## Case presentation

A 32-year-old male from Guayaquil presented to the Emergency Department with pyrexia of unknown origin, severe cephalalgia, emesis, and diarrhea persisting for seven days. The patient also presented with icterus, epistaxis, asthenia, and adynamia. He attributed his symptoms to consuming meals from local street vendors, which he observed to be in suboptimal hygienic conditions.

Upon admission, the patient's condition stabilized, and he was subsequently transferred to the Intermediate Care Unit on the first day. Initially hemodynamically stable, the patient later developed generalized icterus and enterorrhagia, requiring a transfusion of red blood cells due to significant blood loss. Notably, the transfusion was administered between the sixth and seventh hospitalization days, following the initial laboratory investigations. Table 1 shows the clinical progression of the patient during his hospitalization, from intermediate-care admission on the first day until the 17th day. The patient presented with symptoms of diarrhea, enterorrhagia, jaundice, and elevated liver enzymes.

**Table 1:** Laboratory results PT:Prothrombin Time; WBC: White Blood Cells; HGB: Hemoglobin; HCT: Hematocrit; ALT (SGPT): Alanine Aminotransferase (Serum Glutamic Pyruvic Transaminase); GGT: Gamma-Glutamyl Transferase; INR:International Normalized Ratio; WBC: White Blood Cells; CRP (QU): C-Reactive Protein (Quantitative Ultrasensitive)

Test	Result	Reference Range
Red Blood Cell Morphology	Normal	-
White Blood Cell Differential: Neutrophils	80%	34-71.1%
White Blood Cell Differential: Lymphocytes	16%	19.3-51.7%
CRP (QU)	15.18	0-0.5 mg/dL
WBC	4.2	3.98-10.04 10³/µL
HGB	7.5	13.7-17.5 g/dL
HCT	22.3	40-54%
Platelets	238	182-369 10³/µL
Sodium	131.4	137-147 mmol/L
Urea	11.69	17-43 mg/dL
ALT (SGPT)	137.31	≤45 U
GGT	556.98	11-61 U/L
WBC	6.7	3.98-10.04 10³/µL
HGB	9.0	13.7-17.5 g/dL
HCT	25.6	40-54%
Platelets	271	182-369 10³/µL
Sodium	131.2	137-147 mmol/L
Urea	12.16	17-43 mg/dL
ALT (SGPT)	120.03	≤45 U
GGT	918.7	11-61 U/L
CRP (QU)	2.48	0-0.4 mg/dL
PT	11.7	11-16 sec
INR	0.88	0.8-1.5
WBC	5.1	3.98-10.04 10³/µL
HGB	10.5	13.7-17.5 g/dL
HCT	31.6	40-54%
Platelets	584	182-369 10³/µL
Sodium	137.2	137-147 mmol/L
Urea	17.35	17-43 mg/dL
ALT (SGPT)	379.88	≤45 U
GGT	871	11-61 U/L

Laboratory tests and pathogen confirmation

Laboratory tests conducted on the second day of hospitalization confirmed a positive blood culture for *S. typhi* (Table 2 shows the control of rectal swab cultures, which were negative for enteropathogenic bacteria and microorganisms with AMR). The isolation, detected using the Vitek automated system (bioMérieux, Marcy-l'Étoile, France) in the hospital's Microbiology Department, is described. On the 14th day of hospitalization, further molecular analysis using reverse transcription polymerase chain reaction (RT-PCR) confirmed the presence of *S. typhi*, *Leptospira* spp., and *C. coli*. RT-PCR testing was conducted on stool samples for *Salmonella* and *Campylobacter*, and on blood samples for *Leptospira*, by the National Institute of Public Health Research (INSPI) on the 10th day. Serological assessments for viral hepatitis and HIV were negative. All findings were validated by INSPI, confirming concurrent infection by these three pathogens.

**Table 2:** Microbiologial results The isolation detected in the Vitek automated system of the Hospital's microbiology area is described. MDR: Multidrug-Resistant

Test	Result
Blood Culture Aerobic	Positive, *Salmonella* ser. *typhi*
Blood Culture Anaerobic	Positive, *Salmonella* ser. *typhi*
Rectal Swab	Negative for enteropathogenic bacteria or MDR pathogens
Blood Culture Aerobic (Control)	Negative after 4 days of incubation
Blood Culture Anaerobic (Control)	Negative after 4 days of incubation

Treatment and clinical management

The patient was initially treated empirically with cefepime (2 g intravenously every 12 hours) for a total of 14 days, starting on the first day of hospitalization. This broad-spectrum antibiotic regimen was intended to address a range of potential bacterial infections, including *S. typhi*. No alternative antibiotic regimen was administered during this period. Additionally, the patient received parenteral nutrition and supportive care throughout his hospitalization, which included management of liver dysfunction and fluid balance.

Following confirmation of the pathogens, including *S. typhi*, *Leptospira* spp., and *C. coli*, the patient did not receive a change in the antibiotic regimen, as the empirical treatment with cefepime was deemed appropriate for the identified pathogens. The patient’s clinical condition showed steady improvement throughout this period, with a significant reduction in jaundice and normalization of liver enzymes. These improvements were observed after the patient’s stay in the Intermediate Care Unit.

Response to treatment

The patient’s response to the empirical cefepime regimen was positive, with notable clinical improvements within the first few days of treatment. The reduction in fever and jaundice was evident by the third day, and liver enzymes began to normalize within the first week. Despite the initial concerns about co-infections, the patient tolerated the cefepime regimen well, with no adverse effects reported. There were no signs of gastrointestinal distress, and the patient’s overall condition stabilized, allowing for transfer to regular hospitalization after 21 days in the hospital.

The patient did not experience any complications related to antibiotic therapy, and no additional antibiotics were required after the first 14 days of treatment. The patient remained stable, and the concurrent infections were successfully managed with the initial empirical therapy.

Challenges in diagnosis and treatment

The delayed confirmation of pathogens and the complexity of co-infections posed significant diagnostic challenges. The four-day delay in confirming *S. typhi* and the molecular confirmation of *Campylobacter* and *Leptospira* on the 10th day required constant monitoring and frequent reassessment. Differential diagnoses considered included viral gastroenteritis, hepatitis, malaria, and dengue. The presence of multiple pathogens complicated management, but timely adjustments to the treatment regimen, based on culture and PCR results, facilitated effective patient care.

This case report has several strengths, including comprehensive diagnostics, timely treatment adjustments, and a detailed clinical progression of co-infections. However, it has limitations: being a single case, it may not be generalizable; diagnostic delays due to infrastructure limitations were not fully explored; and there is a lack of long-term follow-up. The report highlights the importance of differential diagnoses and AMR, but it could provide more detailed reasoning. Ethically, informed written consent was obtained from the patient, which was particularly crucial given that diagnosing the co-infection with three pathogens was only possible after referral for a febrile icterohemorrhagic study at the National Reference Center.

## Discussion

Concurrent co-infection with *S. typhi*, *Leptospira* spp., and *C. coli* presents a rare and challenging diagnostic and therapeutic scenario, particularly in communities with inadequate healthcare resources (a situation exacerbated by the COVID-19 pandemic) [1-9]. This concern is amplified among local populations and foreign tourists who are at risk of contracting infections from unsanitary food practices at establishments serving "typical cuisine" or from direct exposure to these resistant microorganisms. In such cases, the inefficacy of both first- and second-line antibiotic regimens complicates the situation, particularly in developing regions where medical diagnostic and therapeutic capacities are limited [2-6,10-13].

Leptospirosis, often contracted through exposure to contaminated water, adds complexity because its symptoms overlap with other febrile illnesses, such as typhoid fever [14-16]. The risk of misdiagnosis or delayed diagnosis is significant, potentially leading to severe complications, such as acute respiratory distress syndrome (ARDS) and septic shock, particularly in resource-constrained settings [15]. Although co-infections involving *Leptospira* spp. and other bacterial pathogens are frequently underreported, emerging evidence indicates that such co-infections may increase the severity of the illness and complicate treatment strategies [1,16].

The detection of *C. coli* in this patient further complicated the clinical picture. While *C. coli* is commonly associated with gastroenteritis, it is increasingly being recognized as an opportunistic pathogen capable of causing severe systemic infections, especially in immunocompromised patients [17-20]. This highlights the need for clinicians to remain vigilant for opportunistic infections in co-infected patients, as failure to recognize and treat all involved pathogens can lead to suboptimal clinical outcomes [17-20].

The increasing presence of AMR in *S. typhi* strains is particularly troubling, given the potential scarcity of effective therapeutic alternatives for MDR or extensively drug-resistant (XDR) strains [10,11,20]. The rise in AMR has become a global health concern, driven not only by inappropriate antibiotic use among healthcare providers, but also by the widespread application of antibiotics outside clinical settings, such as in aquaculture, livestock farming, and the poultry industry [20].

Early diagnosis and timely treatment of infections caused by *Leptospira*, *Campylobacter*, and *Salmonella* are crucial for preventing life-threatening complications, such as renal failure, hepatic dysfunction, Guillain-Barré syndrome, pericarditis, and septicemia. Delays in management can significantly exacerbate these outcomes and increase the risk of long-term health consequences and fatalities. Co-infections with these pathogens present an even greater challenge, as the simultaneous presence of multiple infections can overwhelm the immune system, complicate treatment protocols, and increase the likelihood of adverse outcomes. Research has consistently shown that populations with limited access to healthcare are particularly vulnerable to such co-infections, underscoring the need for prompt intervention to reduce morbidity and mortality.

It is important to highlight that, despite the presence of *Leptospira* spp. in the concurrent infection, the patient did not develop acute renal failure, nor did he require renal replacement therapy. This is a notable finding, especially in the context of Weil's syndrome, a severe complication of leptospirosis typically associated with renal failure. This outcome emphasizes the effectiveness of the initial treatment and the importance of timely management in preventing serious renal complications, particularly in patients with co-infections that may complicate the clinical course.

## Conclusions

This case underscores the diagnostic and therapeutic challenges posed by co-infections with *S. typhi*, *Leptospira *spp., and *C. coli* in resource-limited settings. It highlights the growing threat of *S. typhi*, especially in the context of emerging AMR, the opportunistic role of *C. coli*, and the increasing likelihood of multiple co-infections in the era of AMR. The case emphasizes the need to enhance awareness of co-infections in clinical practice and the importance of preventive strategies, particularly in developing countries. Public health measures aimed at infection prevention are critical, especially in areas with poor sanitation.

Moreover, the case reinforces the need for increased surveillance and research into the pathogenicity of these pathogens to improve clinical outcomes. A multidisciplinary approach, incorporating broad-spectrum antibiotics and supportive care, is essential for patient recovery. However, it is also crucial to equip healthcare units with more effective diagnostic tools for early co-infection detection, particularly in endemic regions. For travelers returning from high-risk areas such as Ecuador, heightened clinical awareness, robust diagnostics, and tailored treatments are key to improving outcomes.
